# Automated quality control for within and between studies diffusion MRI data using a non-parametric framework for movement and distortion correction

**DOI:** 10.1016/j.neuroimage.2018.09.073

**Published:** 2019-01-01

**Authors:** Matteo Bastiani, Michiel Cottaar, Sean P. Fitzgibbon, Sana Suri, Fidel Alfaro-Almagro, Stamatios N. Sotiropoulos, Saad Jbabdi, Jesper L.R. Andersson

**Affiliations:** a Wellcome Centre for Integrative Neuroimaging - Oxford Centre for Functional Magnetic Resonance Imaging of the Brain (FMRIB), University of Oxford, UK; b Sir Peter Mansfield Imaging Centre, School of Medicine, University of Nottingham, UK; c Department of Psychiatry, University of Oxford, UK; d Wellcome Centre for Integrative Neuroimaging - Oxford Centre for Human Brain Activity (OHBA), University of Oxford, UK; e National Institute for Health Research (NIHR) Nottingham Biomedical Research Centre, Queens Medical Centre, Nottingham, UK

**Keywords:** Diffusion MRI, Quality control, Movement, Susceptibility, Eddy current

## Abstract

Diffusion MRI data can be affected by hardware and subject-related artefacts that can adversely affect downstream analyses. Therefore, automated quality control (QC) is of great importance, especially in large population studies where visual QC is not practical. In this work, we introduce an automated diffusion MRI QC framework for single subject and group studies. The QC is based on a comprehensive, non-parametric approach for movement and distortion correction: FSL EDDY, which allows us to extract a rich set of QC metrics that are both sensitive and specific to different types of artefacts. Two different tools are presented: QUAD (*QUality Assessment for DMRI*), for single subject QC and SQUAD (*Study-wise QUality Assessment for DMRI*), which is designed to enable group QC and facilitate cross-studies harmonisation efforts.

## Highlights

• Two tools to automatically perform QC of diffusion MRI data.
• Automated generation of single subject reports for visual inspection and database.
• Group databases and reports allow to compare subjects within and between studies.
• Categorical and continuous variables can be used to update the reports.

## 1 Introduction

Diffusion MRI (dMRI) is a powerful tool for probing the in vivo microstructural architecture of the brain (Basser et al., 1994; Bastiani and Roebroeck, 2015; Le Bihan, 1995; Lerch et al., 2017). Several dMRI-based techniques have been developed to estimate fibre orientation (e.g., Basser et al., 1994; Bastiani et al., 2017; Behrens et al., 2003; Tournier et al., 2007), white matter tracts (Behrens et al., 2007; Catani and Thiebaut de Schotten, 2008) and scalar measures that are sensitive to axonal density and white matter integrity (Assaf and Basser, 2005; Zhang et al., 2012).

However, dMRI data acquisition and processing present several challenges. Diffusion weighted datasets are often characterised by low signal-to-noise (SNR) and contrast-to-noise (CNR) ratios, and are frequently corrupted by multiple artefacts. Diffusion MRI data are typically acquired using spin-echo echo-planar-imaging (SE-EPI) sequences. Because of the small bandwidth along the phase encoding (PE) direction of such sequences they are very sensitive to off-resonance fields. A typical bandwidth in the PE-direction is of the order of 10 Hz/pixel, which means that even a (very small) off-resonance field of 10 Hz leads to a 1 pixel displacement.

The off-resonance field in a diffusion-weighted image has two distinct causes. The first is the object (head) itself that disrupts the main magnetic field in a non-trivial way. This is known as a susceptibility-induced off-resonance field (Jezzard and Balaban, 1995). The second is the strong and rapidly switching diffusion encoding gradients that induce eddy currents (EC) in conductors inside the bore. These fields are known as eddy current-induced off-resonance fields (Jezzard et al., 1998). The former type of off-resonance field is as a first approximation constant during a diffusion scan, while the second is different for each sampled diffusion encoding orientation.

Despite using such fast sequences, total acquisition time can be long. E.g., in the Human Connectome Project (HCP) project (Van Essen and Ugurbil, 2012) the total scan time to acquire the dMRI dataset was ∼55 min (Sotiropoulos et al., 2013b). Such long times increase the risk, and the magnitude, of subject movement during the acquisition. Movement is a particular problem in dMRI since it can lead to signal dropout (Wedeen et al., 1994) in addition to gross movement effects (i.e., rotations and translations).

If these artefacts are not properly corrected any model-based parameters derived from the data will be affected (Pierpaoli, 2011). Moreover, residual artefacts might lead to severe biases in subsequent analyses (Yendiki et al., 2014) that can affect group comparisons.

Diffusion MRI data quality can be assessed by visual inspection of each acquired volume. When a volume is deemed to be unusable, it can be removed from the dataset and when a dataset is unusable it can be removed from the study. However, the effects of the different artefacts can be subtle and difficult to visualise across the whole 4-dimensional dataset, which makes visual quality control both non-quantitative and subject to examiner bias. To rely purely on visual QC becomes impractical (if, at all, possible) for large group studies (Alfaro-Almagro et al., 2017). Therefore, there is an emerging need for automated QC procedures that can accurately and with high sensitivity flag up potential problems for subsequent visual inspection.

Few solutions have been published so far on this issue. Phantoms have been used to setup a framework for automated quality control and optimise DTI parameters (Hasan, 2007). Various dMRI data processing tools have been combined to extract useful quality control metrics and perform automated QC (Liu et al., 2010; Oguz et al., 2014). However, currently available tools differ in their capability for detecting most dMRI-related artefacts and, therefore, QC indices derived from them lack a clear consensus (Liu et al., 2015). Generalizability of current QC indices is also limited. Some studies have tested different metrics in big cohorts and between sites (Kochunov et al., 2017; Lauzon et al., 2013; Roalf et al., 2016), but none has tried to assess the accuracy of such metrics across different studies using different data acquisition protocols.

**Fig. 1:**
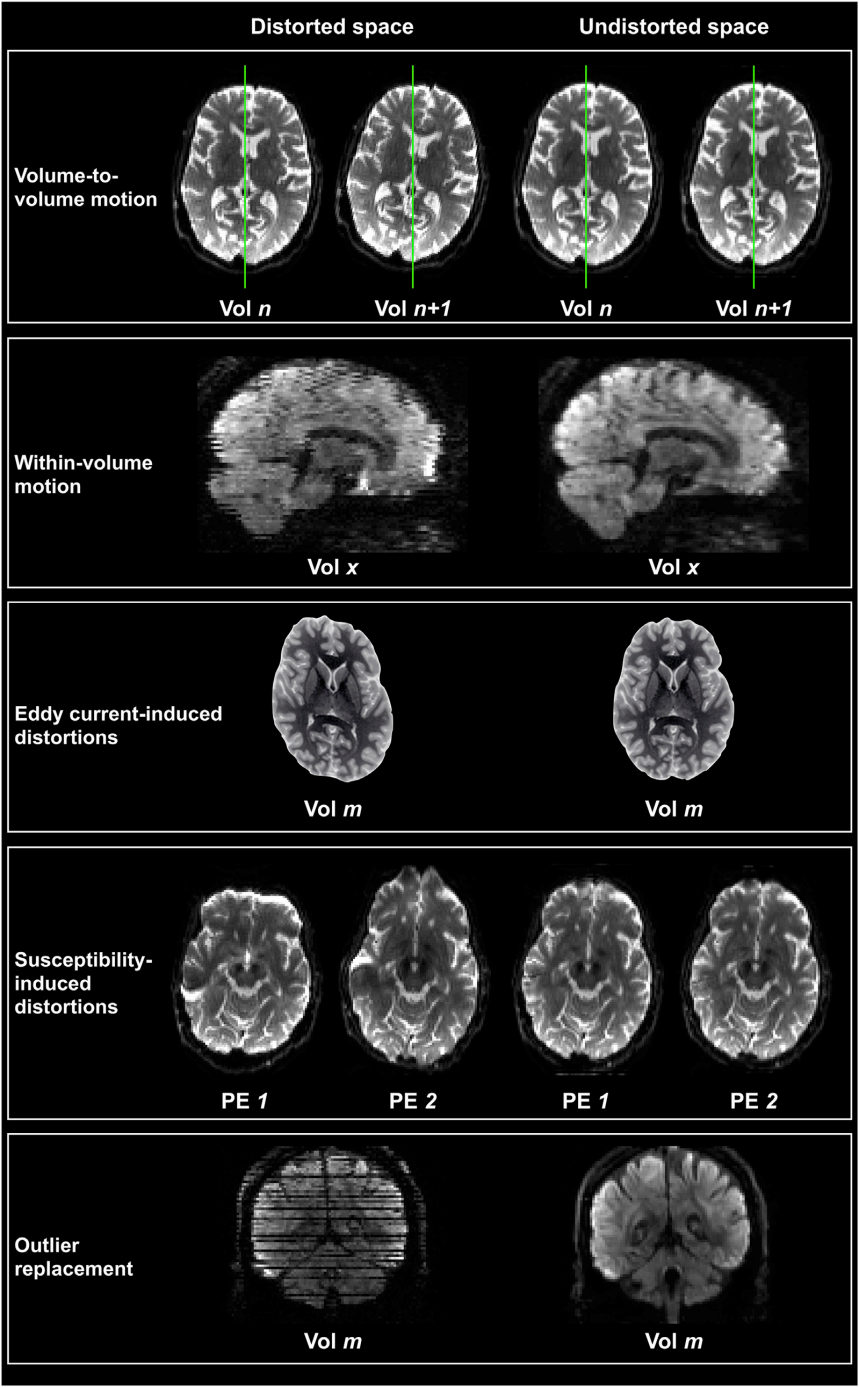
Overview of the EDDY framework for distortions and motion correction. Raw data in distorted space are brought to artefact-free undistorted space. Motion parameters are estimated and used to correct both between and within volumes displacement. Both eddy currents and susceptibility-induced off resonance fields are used to correct for geometric distortions. Using a Gaussian process to describe the 4D data, outlier slices (i.e., slices affected by severe signal dropout) are detected and replaced with their predictions. To illustrate the effects of eddy currents-induced distortions, T2 weighted images have been manually skewed.

In this work, we present an automated dMRI QC framework based on the FSL EDDY tool (Fig. 1; Andersson and Sotiropoulos, 2016). EDDY is a comprehensive pre-processing framework that uses a Gaussian Process (GP) to predict undistorted data, to which the actual observed images can be aligned (Andersson and Sotiropoulos, 2015). It can estimate and correct for volume-to-volume movement and off-resonance fields (Andersson and Sotiropoulos, 2016), signal dropout caused by movement during the diffusion encoding (Andersson et al., 2016), within-volume movement (Andersson et al., 2017) and movement-induced changes of the susceptibility-induced off-resonance field (Andersson et al., 2018). In addition to correcting for these effects, the output from this framework offers a rich description of the off-resonance and subject movement effects present in the uncorrected data.

In addition, the GP allows us to quantify the diffusion angular CNR (the diffusion related variance vs the noise variance) in a way that is independent of any model commonly used to analyse diffusion data. Importantly, this CNR measure is completely data driven without any need for model or model order selection. This allows us to propose QC metrics that are both sensitive and specific to different artefacts.

Here we present two different tools that use the output from the FSL pre-processing tools to automatically perform QC. The first, QUAD (*QUality Assessment for DMRI*), automatically generates quantitative single subject reports in a largely graphical format convenient for visual inspection. Additionally, it stores the quality control metrics for each subject in a format that is convenient for further processing. The second tool, SQUAD (*Study-wise QUality Assessment for DMRI*), is designed to facilitate group studies and harmonisation efforts. It reads all the single subject outputs from QUAD, generates study-wise reports and, optionally, enters these into a database that can be shared with the imaging community. Moreover, SQUAD can optionally update the single subject reports, indicating how the subject's dataset compares to other data, using either a study-specific group database or a pre-generated database obtained from a different dataset. Lastly, SQUAD also allows to report QC indices based on user-provided grouping variables.

## 2 Methods

### 2.1 Quality control metrics

Our automated QC framework relies on EDDY (Andersson and Sotiropoulos, 2016), a comprehensive FSL-tool (Smith et al., 2004) for pre-processing of dMRI data. One of the inputs to EDDY is a brain mask, which we mention here since it is used by the QC-tools. The following EDDY-derived metrics are used in the QC tool:

● *Volume-to-volume motion*: A total of 6 parameters are used to quantify subject motion *between* different volumes. These consist of 3 translations and 3 rotations around the x, y and z axes. A summary measure of “total motion” is then calculated as the average voxel displacement across all voxels within the brain mask. Such voxel-wise average voxel displacement summarises both rotations and translations at each voxel with a single scalar. Absolute (w.r.t. a reference volume) and relative (w.r.t. the previous volume) total motions are calculated for each volume. We also derive summary QC metrics by averaging absolute and relative motion estimates over all volumes.
● *Within-volume motion*: A recent extension of EDDY is able to realign slices within a single volume affected by motion artefacts (Andersson et al., 2017). Six motion parameters (3 translations, 3 rotations) are estimated for each slice or block of simultaneously acquired slices (if simultaneous multi slice/multiband is used). We derive summary QC metrics by averaging the standard deviation of each parameter calculated across the slices/groups of a volume, which quantifies the amount of subject movement *within* that volume.
● *Eddy current-induced distortions*: In EDDY, the eddy current-induced off-resonance field is modelled using a low order polynomial (typically second order). We derive QC metrics by computing the standard deviation of the three coefficients of the first order terms across the whole acquisition. This is a measure that quantifies the volume-to-volume variability in EC distortions across the scan. It reflects a combination of the magnitude of the EC-induced off-resonance fields and the bandwidth in the PE-direction.
● *Susceptibility-induced distortions*: To correct for susceptibility-induced distortions, EDDY uses the off-resonance field estimated by the FSL TOPUP tool (Andersson et al., 2003). The field can be converted into a voxel displacement map. We derive a QC metric reflecting the amount of geometric distortions due to differences in tissue susceptibility by computing the standard deviation of voxel displacement values within the brain mask.
● *Outlier replacement*: Subject motion during the acquisition of a single slice (or group of simultaneously acquired slices) can result in signal dropouts (Fig. 1). These are automatically detected within EDDY, and optionally replaced by the GP predictions We extract QC metrics by computing the percentage of slices classified as outliers per volume and across the whole dataset. They are also grouped by b-value and/or phase encoding (PE) direction.

Two additional quality control metrics are extracted from the dMRI dataset. When multiple b0 volumes (i.e., volumes with no diffusion-weighting) have been acquired, the voxel-wise signal-to-noise ratio (SNR) is calculated as the mean divided by the standard deviation of the signal. The EDDY QC tools calculate the average SNR across all voxels within the brain mask to give a summary measure of the overall quality of the dataset.

To assess the quality of the diffusion-weighted volumes, we use the predictions from the EDDY model. EDDY calculates a voxel-wise angular contrast-to-noise ratio (CNR) image as the ratio of the standard deviation of the predicted signal and the standard deviation of the residuals. Both standard deviations are computed across all the sampled directions sampled on the same b-value shell. A summary CNR measure is then calculated as the average CNR for each b-shell across all the voxels within a user-specified brain tissue mask. This quantifies the amount of angular contrast to noise. Table 1 gives an overview of all the EDDY-derived quality metrics.

**Table 1:**
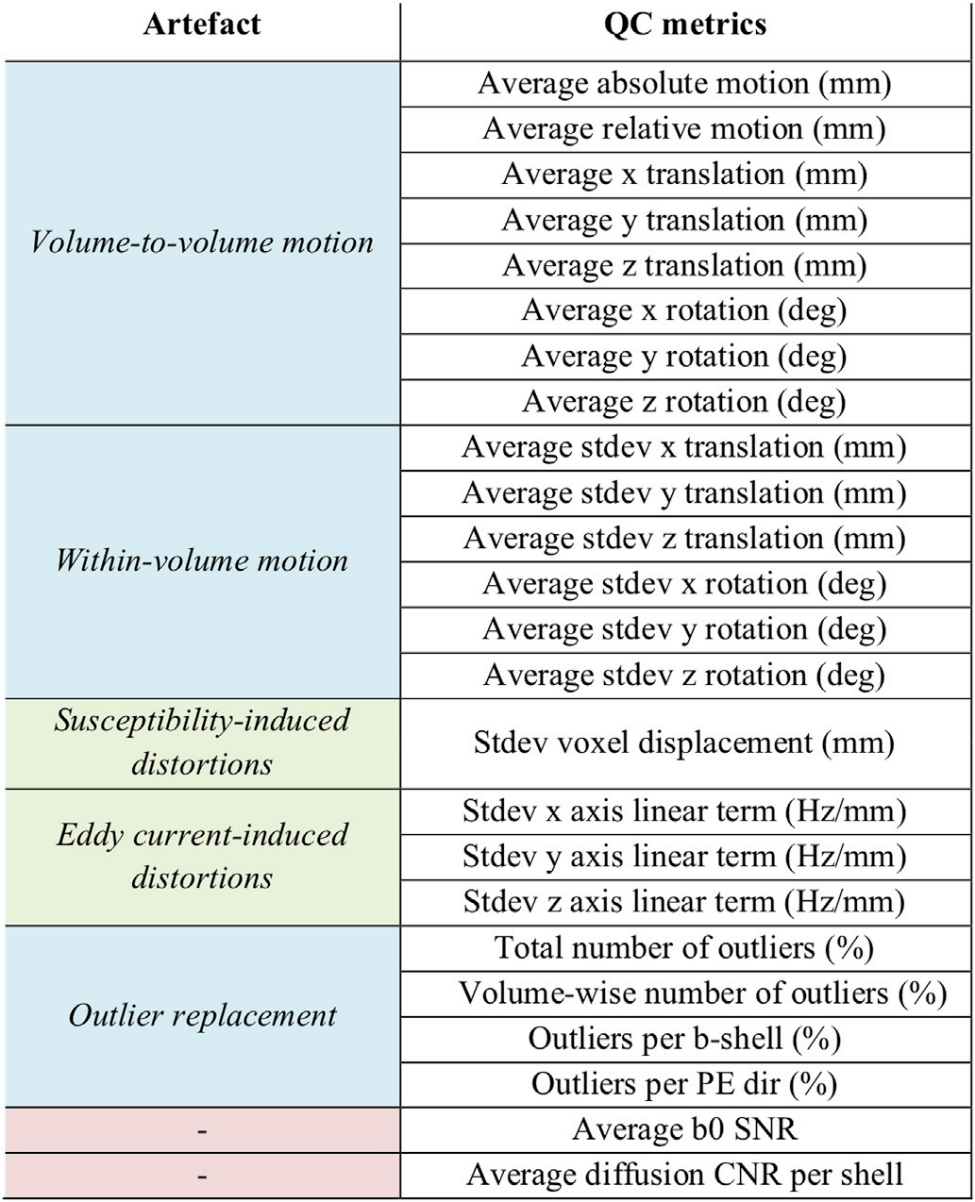
Overview of QC indices. The EDDY QC framework generates and stores relevant quality control metrics from different EDDY outputs and generates summary figures. All motion-related metrics are reported relative to image space (i.e., not in scanner space). Cell colours indicate classes of QC metrics: motion-related (blue), distortions-related (green), SNR and CNR (red).

### 2.2 Single scan/subject quality control

After EDDY has been used to pre-process a dMRI dataset, QUAD can be used to extract the quality control metrics described in the previous section. The availability of those metrics will depend on the exact options that EDDY was run with. This is determined by QUAD based on what EDDY output files are present. For example, metrics related to outlier slices are not calculated when the corresponding EDDY option has not been used.

QUAD generates a folder containing two main QC output files for each subject. One is a report in PDF format containing plots and tables aimed at helping a human observer to get an “at a glance” picture of the overall quality of the data set. The other is a database entry containing all the quality assessment metrics stored in a JavaScript Object Notation (JSON) text file.

### 2.3 Study/population-wise quality control

Quality control at the group level can be performed using the tool SQUAD. The main input to this tool is a list of single-subject QC folders, and the JSON files therein, generated by QUAD. It reads the subject-wise QC files and calculates summary statistics of all the metrics described above. Its basic outputs consist of a folder containing a study-wise report stored in PDF format and one JSON text file. The report PDF contains plots of distributions of all the quality control metrics across all the subjects. The JSON file stores the study-wise database of quality control metrics obtained from all the individual subjects.

The study-wise tool has been designed to allow flexibility in terms of how the QC metrics are visualised in the PDF report. One can for example provide auxiliary measures, categorical and/or continuous, which SQUAD will use to provide additional plots as part of the PDF report. When using a categorical variable, the distributions of each QC metric is displayed as separate violin plots for each category. When providing a continuous variable, a scatter plot of each QC metric is generated. This can give an immediate impression of the extent to which for example subject movement is correlated with a variable that will later be used as an explanatory variable in subsequent analysis.

### 2.4 Single subject quality control in the context of a study/population

Importantly, SQUAD can also update all the single subject reports provided using a QC database generated by SQUAD. This can be the database obtained from the same study, which allows the user to visualise each subject's QC metrics in the context of the group. Outlier subjects for each QC metric are automatically flagged and their individual QC reports are updated using a traffic light colouring system, yielding an even more graphic and immediate picture of the quality of an individual data set. Each QC metric is flagged as a moderate (severe) outlier if it is more than one (two) standard deviation away from its group average.

SQUAD can also update individual QC reports using a QC database from another study with, potentially, a different imaging protocol. This means that it can be used as a tool for piloting new protocols by giving an immediate comparison to the data quality of an existing protocol, such as for example the HCP or the UK Biobank.

### 2.5 MRI data analysis

We selected data from three large population studies to showcase the automated QC tools. Data from 100 subjects were selected randomly from the UK Biobank (Miller et al., 2016), the young adult Human Connectome Project (YA HCP) (Van Essen and Ugurbil, 2012) and the Whitehall II Imaging Sub-study (Filippini et al., 2014). These three studies cover a range of acquisition parameters (e.g. high vs low spatial resolution or single vs multi-shell). Details on the data acquisition protocols are provided elsewhere (Filippini et al., 2014; Miller et al., 2016; Sotiropoulos et al., 2013b). For this work, we used data from a subset of participants from the Whitehall II Imaging Sub-study, who received an additional dMRI scan with acquisition parameters which deviate from those listed in the protocol paper. Table 2 summarises the main acquisition parameters that impact the extracted quality assessment indices.

**Table 2:** Summary of dMRI data acquisition parameters for the three large population studies used to test the EDDY QC tools. Numbers of acquired diffusion weighted directions are specified for each shell. The numbers between brackets refer to additional volumes acquired for the second phase encoding direction using the same b-value.

	UK Biobank	YA HCP	Whitehall II
Voxel size (mm)	2.0 × 2.0 × 2.0 mm	1.25 × 1.25 × 1.25 mm	1.5 × 1.5 × 1.5 mm
MRI Scanner	3T Siemens Skyra	3T Connectom Skyra	3T Siemens Prisma
b-values (s/mm^2^)	1000, 2000	1000, 2000, 3000	1500
No. dw directions	50 (0), 50 (0)	90 (90), 90 (90), 90 (90)	60 (60)
No. b0 volumes	5 (3)	18 (18)	5 (5)
PE directions	AP (PA)	LR (RL)	AP (PA)
Volume size	104 × 104 × 72	144 × 168 × 111	128 × 128 × 84

Pre-processing was run on the data to correct for eddy current and susceptibility induced distortions, slice dropouts, bulk motion and within volume motion.

## 3 Results

**Fig. 2:**
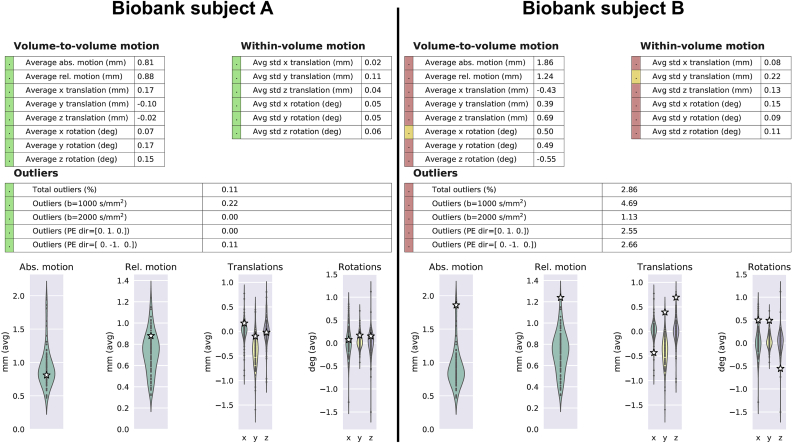
Example summary tables from two single subject reports generated using QUAD. Top row: individual QC metrics are flagged as outliers by SQUAD based on their within-study distributions. The traffic light colouring scheme indicates that QC metric is less than a standard deviation away from the mean (green), between one and two standard deviations (yellow) or more than two standard deviations away (red). Bottom row: example distributions (across subjects) of the between volumes motion parameters are shown using violin plots. The individual subject corresponding to this report is marked with a white star.

The EDDY QC tools were used to generate single subject and study-wise reports and databases. Fig. 2 shows two examples of single UK Biobank subject reports generated by QUAD and updated by SQUAD. The tools automatically detected that EDDY was run using slice outlier detection and replacement and within-volume movement correction. After the updating of the single subject reports by SQUAD, single indices can be flagged as outliers. In the cases shown in Fig. 2, this is done by comparing individual subject metrics against the distribution obtained from the same study. SQUAD generates visual summaries of each distribution and puts each subject into context by adding extra pages to the single subject report.

**Fig. 3:**
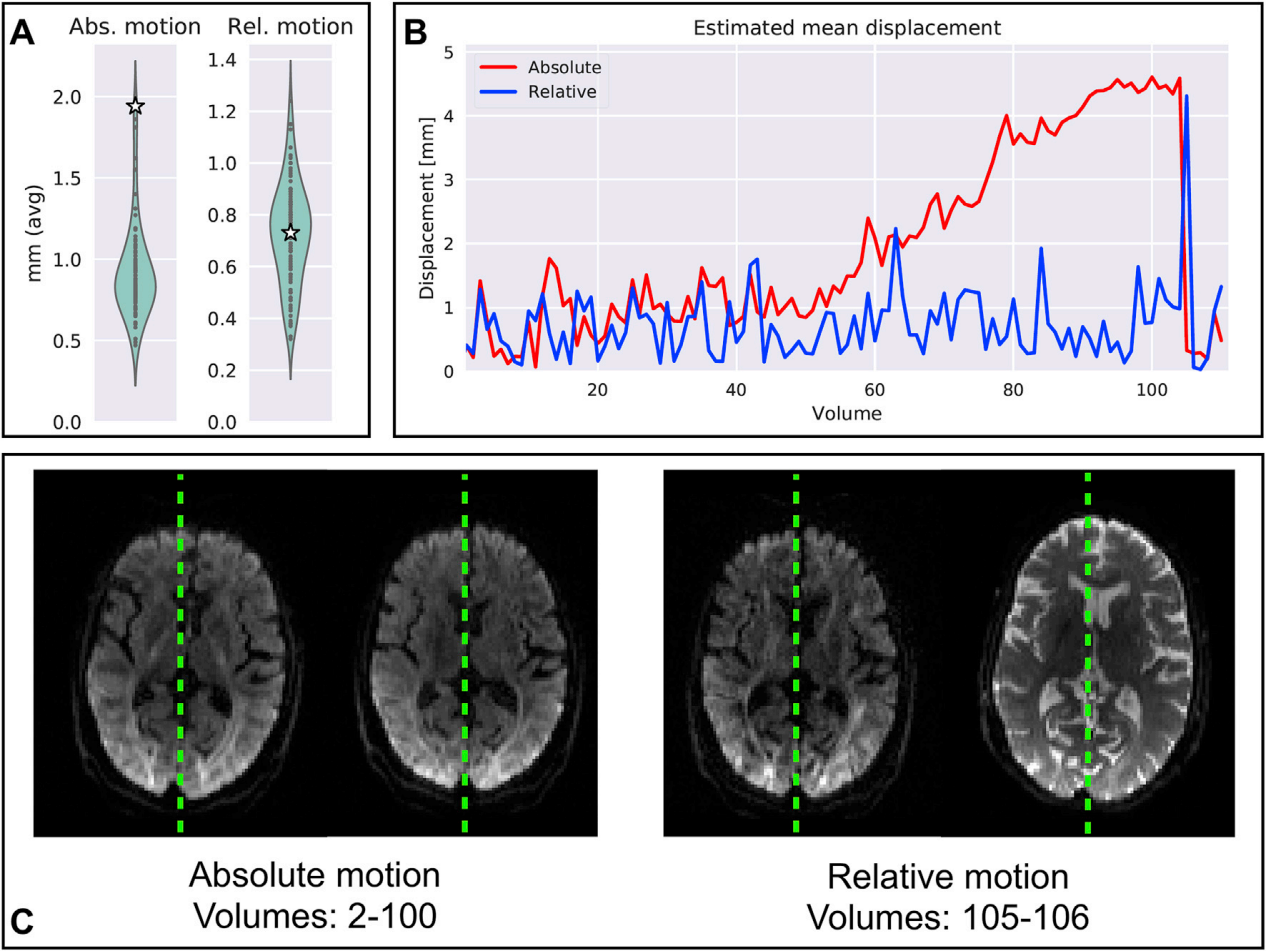
Single UK Biobank subject with high degree of estimated between-volumes motion. A) In the updated single subject report, the estimated mean displacements (absolute and relative) are plotted against the study-wise distributions. B) From the displacement time-courses visualised in the single subject report, the volumes where the subject has moved the most can easily be extracted and compared. C) Example axial slices prior to correction (not present in the single subject report) from four volumes are provided in the bottom row, where the displacements due to severe absolute and relative motion are visible. Green lines are put as reference to the brain's midline.

Fig. 3 shows an example subject for whom between-volume average absolute motion has been flagged as an outlier by SQUAD. Looking at the estimated time courses of average and relative volume-wise motions, it is possible to identify volumes that show the highest amount of displacement. This can be done both across the whole acquisition, looking at the average absolute motion, or between two consecutive volumes, looking at the relative motion.

**Fig. 4:**
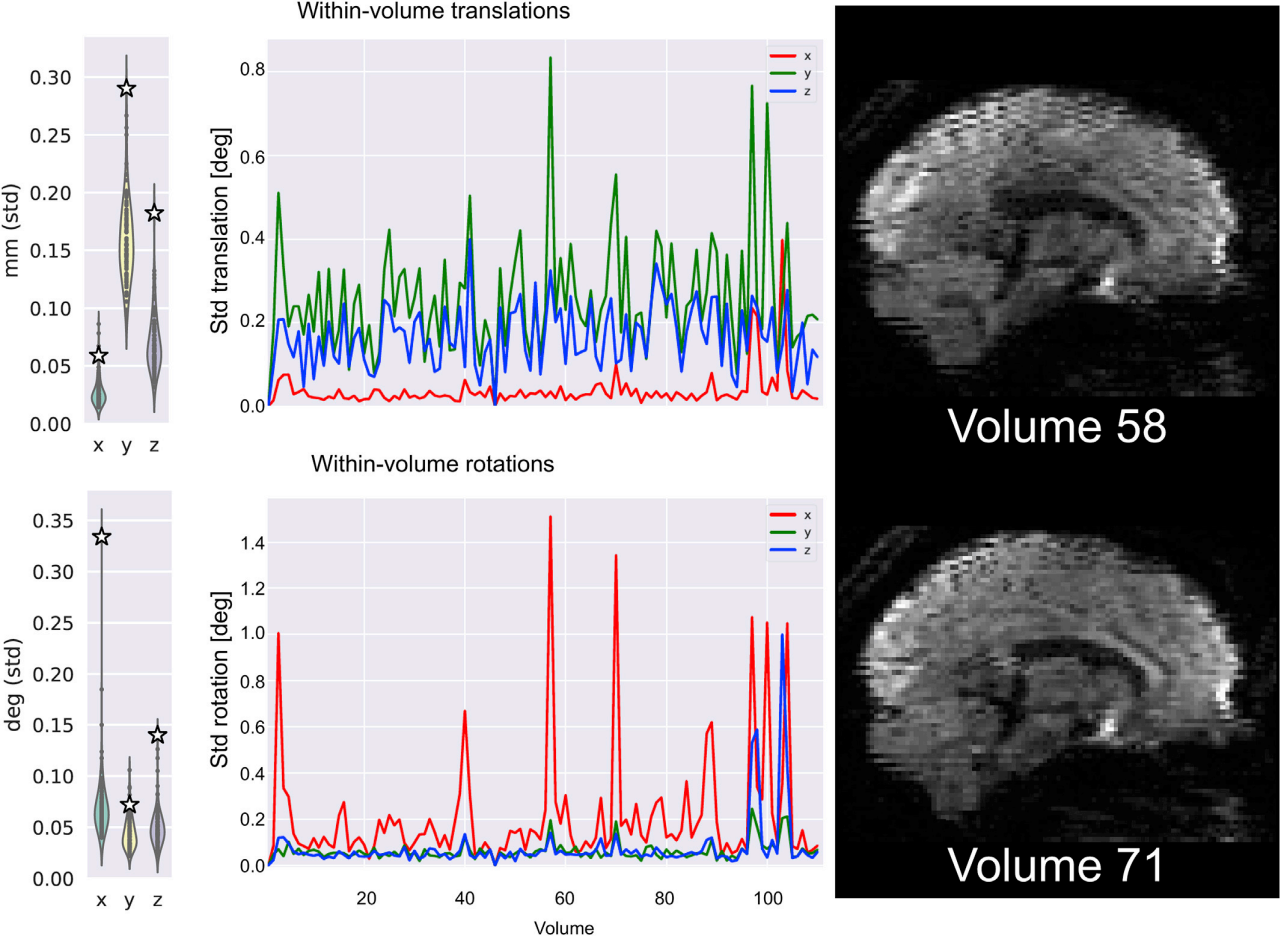
Single subject with high degree of estimated within-volume motion. This subject's mean standard deviation of the estimated motion parameters (3 translations and 3 rotations) are plotted against their study-wise distributions in the updated single subject report. From the parameters' time-courses shown in the single subject report, two example volumes where the subject has moved the most can easily be extracted and inspected. The two sagittal views prior to correction (not present in the single subject report) show that consecutive slices are misaligned in the distorted, i.e., uncorrected original dataset.

Fig. 4 shows an example subject with high degree of within-volume motion. Looking at the distributions of the relevant quality control metrics, the subject can be easily identified. Further visual assessment can be done by looking at the time courses of the 6 estimated indices. The figure shows two example volumes characterised by significant amount of within volume translations along the y axis and rotations around the x axis.

**Fig. 5:**
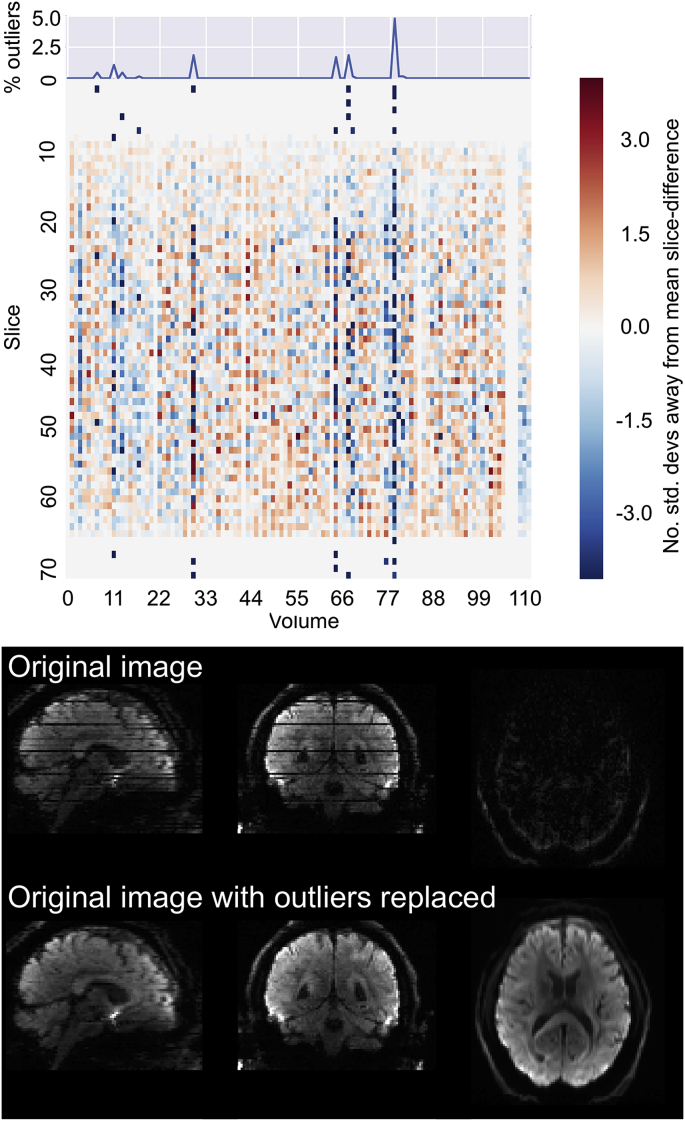
Assessing frequency and distribution of signal dropout outliers. In the single subject report, volume-wise percentage of detected outlier slices is plotted on top of a heatmap showing the number of standard deviations away from the mean slice difference. Axial and sagittal views (not present in the single subject report) show an example volume (no. 78) before (top row) and after (bottom row) outlier detection and replacement.

High degree of motion during the diffusion encoding can result in signal dropouts in different slices. Fig. 5 shows that, using QUAD, it is possible to identify volumes that are severely affected by signal dropouts. These can be identified by looking at the volume-wise percentage of slices that were classified as outliers by EDDY. Further visual inspection of affected volumes can be performed to assess the success of the outlier replacement step. One of the outputs of EDDY is the original images (not corrected for any distortions, movements, etc.), but where the slices that were deemed as outliers have been replaced by the GP predictions. Fig. 5 shows a raw volume compared to the same one where outliers have been detected and replaced.

**Fig. 6:**
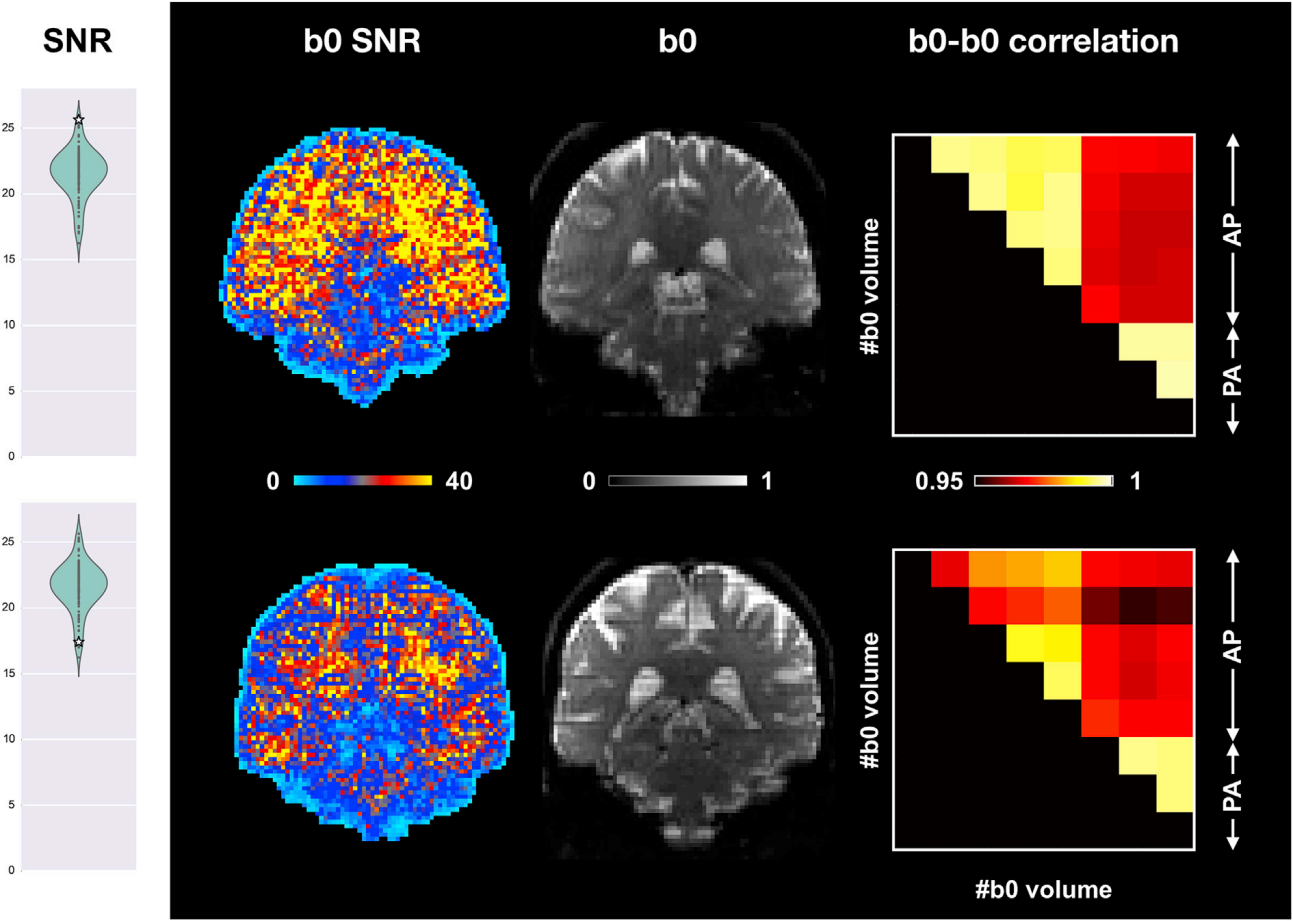
Comparison of two UK Biobank subjects based on their b0 SNR. Average SNR values (white stars overlaid on violin plots) and maps show clear differences between the two subjects. A further between-volumes correlation analysis reveals residual issues after pre-processing of the second subject (bottom row). Coronal sections of the second b0 volume show spin-history effects for the second subject. Coronal sections and correlation matrices are not shown in the QC reports. For the correlation matrices, AP (Anterior->Posterior) and PA (Posterior->Anterior) refer to the two phase encoding directions used for data acquisition.

Fig. 6 shows two example subjects with high and low b0 SNR. Low values of SNR hints at residual issues that could not be fully corrected during pre-processing. This becomes clearer looking at the between b0 volumes correlations. These show that the second b0 volume in the AP phase encode direction is driving the SNR down. That is most likely a consequence of spin history effects caused by subject movement during this or the preceding volume.

**Fig. 7:**
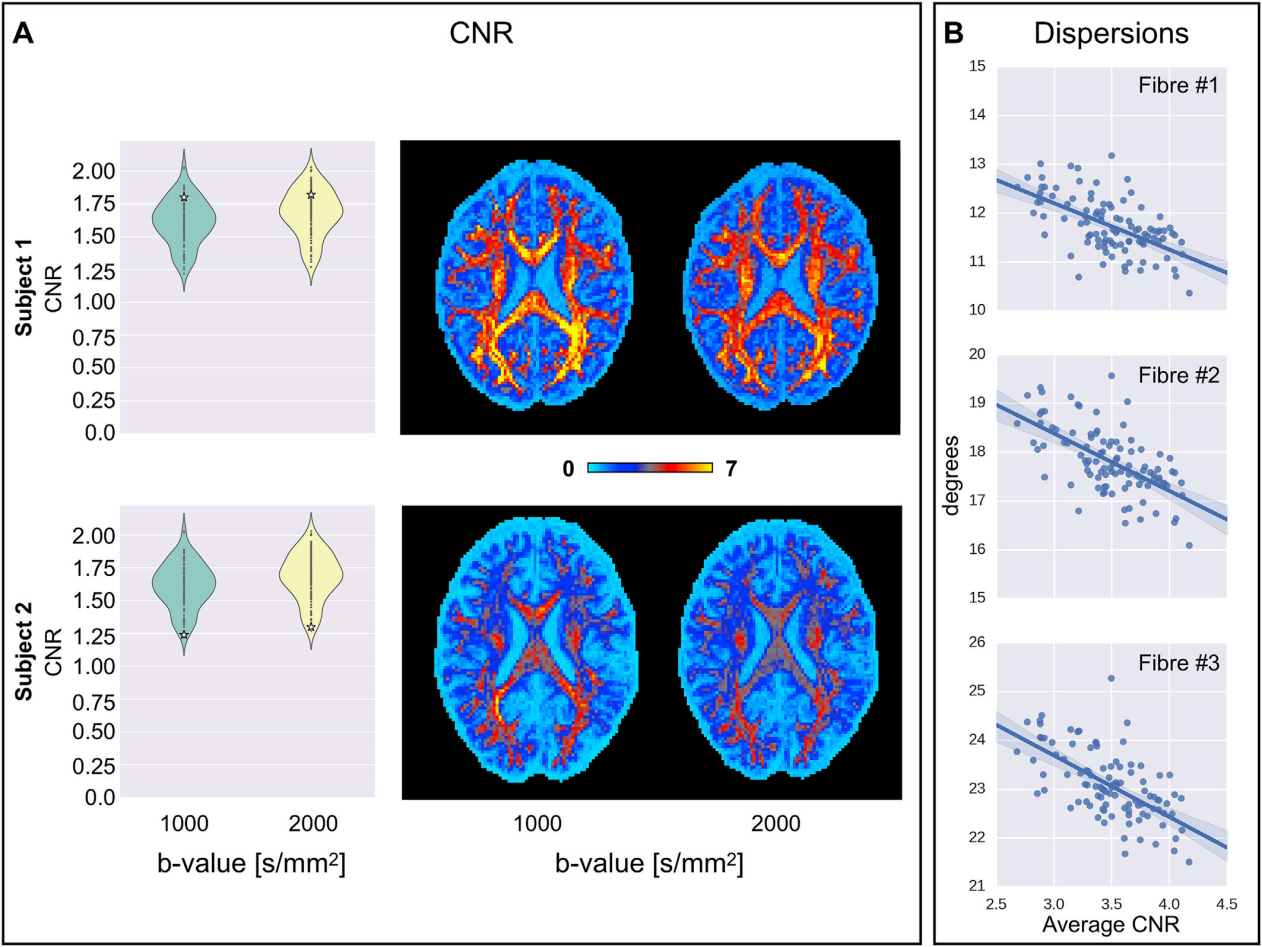
A) Comparison of two UK Biobank subjects based on their diffusion CNR. Average CNR values (white stars overlaid on violin plots) and axial slices show clear differences between the two subjects. B) A further regression analysis highlights the dependency of estimated white matter fibre dispersion on CNR for 100 UK Biobank subjects. Average white matter CNR accounts for both the b1000 and b2000 shell CNR. Fibre dispersion is reported in degrees. Each point on the scatterplots represents a single UK Biobank subject.

To assess the benefits of having a high CNR dataset, we investigate our ability to characterize multiple fibres in a single voxel and how that depends on the CNR using the 100 UK Biobank subjects. For that purpose, we fit up to three fibre compartments in each voxel using the multi-shell extension of the ball-and-stick model (Behrens et al., 2007; Jbabdi et al., 2012). Fig. 7 shows that, for each fibre compartment, a higher CNR reduces the dispersion of the estimated fibres. The dispersion is a scalar value that represents the uncertainty on the fibre orientation encoded in the posterior distribution. It is defined as $1-\lambda_{1}$, where $\lambda_{1}$ is the largest eigenvalue of the average dyadic tensor implied by each sampled orientation. Averaged white matter fibre dispersion values were obtained using a white matter mask comprising only voxels with high fibre complexity. A white matter mask from T1 anatomical space was initially transformed into diffusion space using FSL's FLIRT (Jenkinson et al., 2002). Then, only those voxels where the volume fraction of the third fibre was higher than 0.05 were retained from each individual white matter mask in diffusion space. This mask was then used to extract average CNR, SNR and fibre dispersion estimates. To further quantify the benefits of having high CNR, we computed the *partial* correlation between the fibre-wise dispersion estimate and the CNR average of both shells after regressing out the average SNR. For each fibre compartment, we found that partial correlations were equal to −0.71, −0.76 and −0.76 (all p < 0.01), respectively. This shows that, a high CNR dataset will yield better estimates of fibre orientations, which will in turn deliver better tractography results.

**Fig. 8:**
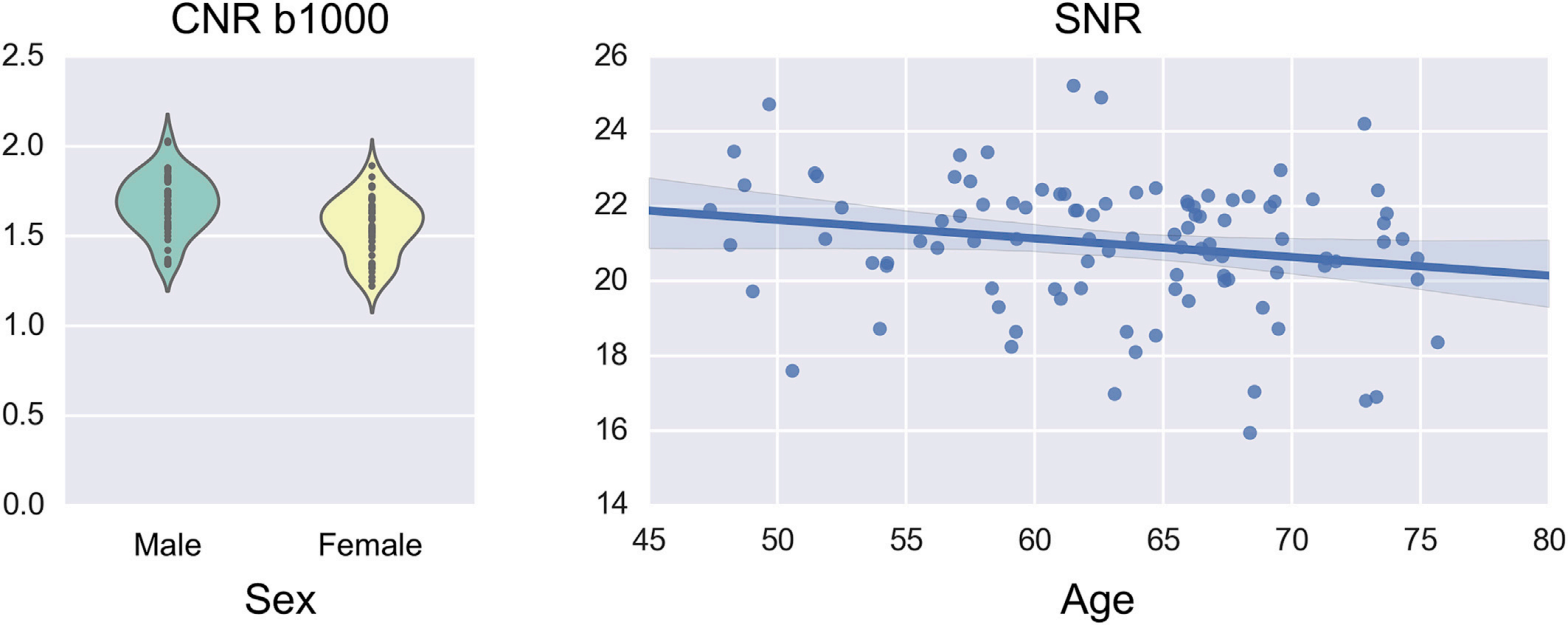
Differences in quality indices using categorical and continuous variables. Left panel shows differences in the distributions of the average CNR for the b1000 shell between females and males. Right panel shows the average SNR plotted against age. Both panels use the 100 analysed UK Biobank subjects.

Quality control is not limited to single subject analysis. General trends in specific quality indices can be visualised using grouping variables. SQUAD accepts as inputs both categorical and continuous grouping variables. When provided, it generates and adds to the group (and single subject) report either a violin plot for categorical or a scatter plot for continuous variables. Examples of such plots are shown in Fig. 8. Plots like those in Fig. 8 would serve as warnings if one were to investigate gender or age differences in subsequent analyses.

**Fig. 9:**
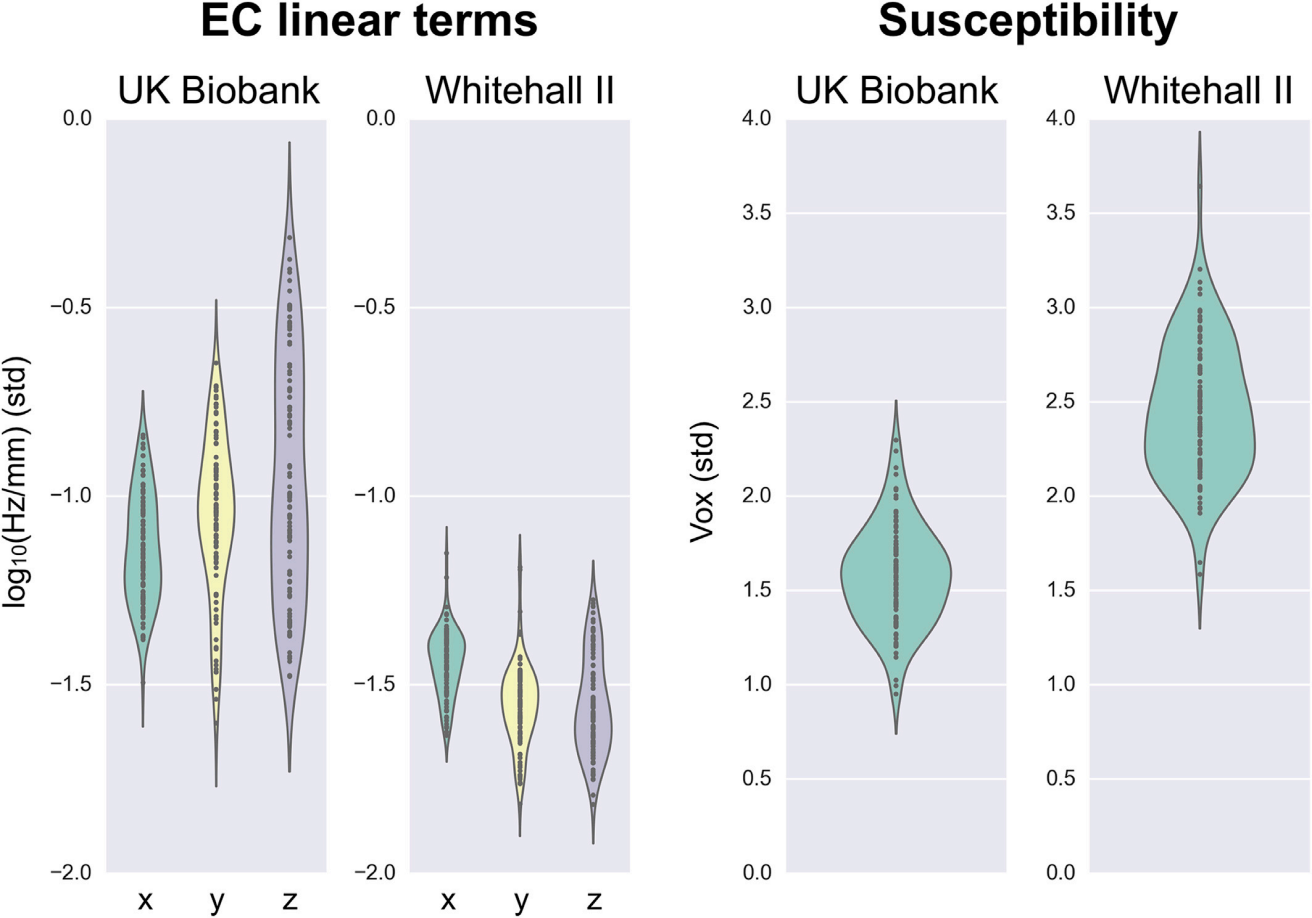
Comparison between amounts of induced distortions by eddy currents and differences in susceptibility in the UK Biobank and Whitehall II imaging studies. Different scanning sites and protocols yields differences in the amount of distortions. All violin plots are added into the group report generated by SQUAD. Axes were rescaled in this figure.

The EDDY QC tools can also be used to compare quality indices between studies to evaluate acquisition protocols and/or sites performances. E.g., the amount of eddy currents and susceptibility-induced distortions can be compared across different protocols. Fig. 9 shows the distribution of their indices for the UK Biobank and the Whitehall II studies. The eddy current-parameters (left panel) reflect both hardware and sequence factors, such as for example Stejskal-Tanner versus twice-refocused diffusion encoding (Reese et al., 2003), that should be accounted for when designing a dMRI experiment. Both the mean and dispersion of the EC-parameters are of interest as the former reflects the overall level of ECs and the second the scan-to-scan reliability of the EC-behaviour. It can be seen in Fig. 9 that the UK Biobank data seems to perform slightly worse than the Whitehall II data on both these counts, which we believe reflects a non-optimal and more variable bed position in the former data set. The susceptibility-induced distortions (right panel) mainly reflect the bandwidth-per-pixel in the phase encode-direction.

**Fig. 10:**
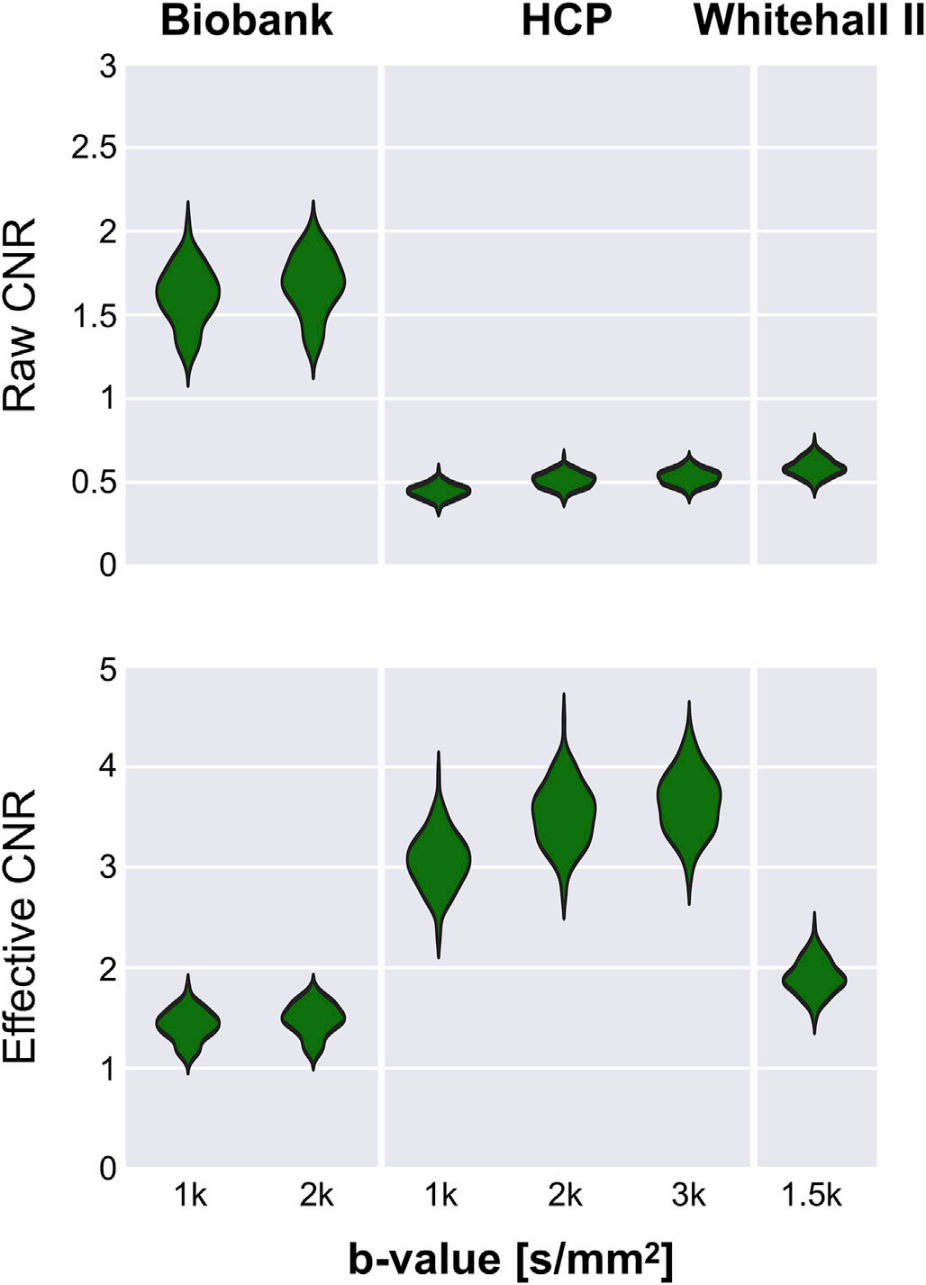
Raw and effective b-shell average CNR distributions for all the three studies. CNR values were averaged within a binary brain mask for each subject. Violin plots show the smoothed histograms from 100 subjects for each study. After rescaling to account for number of acquired volumes for each b-shell and voxel size, effective CNR leads to more comparable results across the different studies.

Care needs to be taken when comparing QC results across studies. Fig. 10 shows the CNR distributions for each b-shell acquired in the three different studies. Raw CNR values would indicate that Biobank data has higher CNR than data from either HCP or Whitehall II. The results change when accounting for differences in voxel sizes and number of acquired volumes for each shell. We, therefore, compute an effective CNR measure by multiplying the raw CNR values by the square root of the number of acquired volumes and dividing it by the voxel volume. Taking into account these two factors leads to the Biobank and the Whitehall II study being more comparable, while the HCP shows the highest average effective CNR. As expected, the CNR also shows a dependency on b-values.

## 4 Discussion

In the present work, we introduce a new automated QC framework based on the FSL EDDY tool. It offers automated QC both at the single subject and the study-wise level using two separate tools named QUAD and SQUAD, respectively. Given the EDDY output, QUAD automatically extracts quality assessment metrics, stores them and generates a report. When multiple QUAD reports are available from the same study, SQUAD reads them to create a study-specific database and generates a study-wise report. Moreover, it can update single subject reports based on any previously generated database and allows metrics to be grouped according to continuous and discrete variables. We think that this automated QC framework can be helpful when designing a new study, or to identify inconsistent and problematic datasets within a study. Crucially, it can be used when assessing very large datasets, such as those used in this work, where visual QC is not practical.

EDDY allows simultaneous correction of susceptibility- and eddy current-induced distortions as well as between and within-volume movement and signal loss caused by subject movement. By incorporating all of these effects into a comprehensive model, several quality control metrics can be derived that reflect potential issues with some datasets. When assessing the quality of each individual dataset against all the others within the same study, our tools automatically flag potential outlier subjects. Importantly, such outliers are defined separately for each individual metric. For example, in the case of a subject with unusually high movement, the average absolute motion metric of such a subject will be flagged as outlier in the updated report. It is then easy for the user to visually inspect that specific subject and decide whether to keep, discard or down-weight the subject or just selectively remove some of the volumes.

It is important to note that many of the metrics reported by QUAD/SQUAD have been estimated and subsequently used to correct the data. In principle, if the correction is good enough, that would mean that even if a subject has moved more than “normal” the data can still be completely usable. The EDDY tool keeps incorporating additional effects of movement into its generative model (Andersson et al., 2016, 2017, 2018; Andersson and Sotiropoulos, 2016) and is hence expected to be able to deal with increasingly problematic subjects. It has for example recently been shown that the outlier replacement is able to largely reverse the effects of movement on estimates of structural (diffusion based) connectivity (Baum et al., 2018).

In that respect, the CNR and SNR measures have a special role in that they tell something of the “outcome” of the (EDDY) pre-processing. One can for example envisage a case where EDDY has failed to accurately estimate subject movement and detect outliers (for example because of too excessive levels of movement). In such a case, the QC metrics based on movement and outlier metrics might not imply anything untoward. In contrast, the SNR and/or the CNR measures would likely be strong outliers that would warrant further manual inspection. Conversely, the movement metrics might indicate an outlier but if EDDY has been run with outlier replacement (Andersson et al., 2016), correction for within volume movement (Andersson et al., 2017) and susceptibility-by-movement correction (Andersson et al., 2018) it may have been able to correct for the effects of movement such that the SNR and CNR of the corrected data is well within the usable range.

It should be noted that any estimate of CNR, *i.e.* ratio between diffusion-induced signal variability and non-related signal variability, will depend on the particular assumptions made. If one for example used a diffusion tensor model to estimate CNR one would severely underestimate CNR in areas of complex anatomy (crossing fibres). The GP used in EDDY has been shown (Andersson and Sotiropoulos, 2015) to be able to model the signal from highly complex areas, so should be able to accurately estimate CNR also in those areas. The GP (*i.e.* the covariance function) is parameterised by a small number (three for single shell data) of hyperparameters estimated directly from the data. Even though the hyperparameters are the same for all voxels the fitting of the GP is dominated by the data so even though the signal from different voxel is vastly different in complexity (e.g. grey matter vs three-way fibre crossings) it is modelled equally well.

Our framework can also be used to assess the quality of a newly acquired dataset against pre-existing databases. This can be useful when optimizing acquisition parameters for a new study. For example, if trying to match a dMRI acquisition protocol to that of the UK Biobank, the user can load the pre-generated Biobank QC database and compare their data against pre-computed metrics. This will show how the proposed protocol compares with respect to a number of metrics such as levels of susceptibility and eddy current-induced distortions, SNR and CNR. Several quality metrics might depend on specific scanning parameters, such as voxel volume, readout bandwidth and imaged volume's size (Sotiropoulos et al., 2013a). These factors need to be accounted for when comparing subjects between studies (Fig. 10). Moreover, considering amount of motion per unit time rather than per volume (as done in this work) may further improve subject comparisons across-studies. We hope our QC metrics may help in this harmonisation effort.

Most studies typically report SNR values when assessing the quality of individual dMRI datasets. Despite this being a very important metric, it is limited as it does not reflect the actual angular contrast available in that specific dataset. From a modelling perspective, such contrast is crucial to estimate fibre orientations or microstructural indices. Therefore, to allow quantitative assessment and comparison of dMRI data, we have introduced a CNR measure. For each b-shell, we compute a voxel-wise CNR map as the ratio between the standard deviation of the predicted data and the standard deviation of the residuals, which can both be obtained when using EDDY. We showed that this metric reflects the level of angular contrast available in the dataset and higher CNR improves the estimation of complex fibre configurations (i.e., more than one fibre compartment in each voxel). Assessing the CNR at different b-values can be useful to design a new study. However, as for SNR, other parameters should be considered when comparing CNR across different studies. We have introduced the concept of effective CNR, that accounts both for differences in voxel sizes and number of acquired volumes.

The code will be publicly released via FSL. The Python code and example QC reports can already be downloaded from: https://git.fmrib.ox.ac.uk/matteob/eddy_qc_release.git. The tools can be easily extended by including more quality control metrics. These will be mainly added based on the progression of the EDDY tool, but others can be added using the current available output.
